# Autologous skin-grafting surgery with novel continuous liquid infusion stent for prevention of esophageal stenosis after complete circular endoscopic submucosal tunnel dissection

**DOI:** 10.1055/a-2268-5590

**Published:** 2024-04-04

**Authors:** Shengzhen Liu, Ningli Chai, Yunjuan Lin, Nanjun Wang, Longsong Li, Nan Zhang, Enqiang Linghu

**Affiliations:** 1 Department of Gastroenterology, The First Medical Center, Chinese PLA General Hospital, Beijing, China

We usually prevent postoperative esophageal stenosis with autologous skin-grafting surgery for complete circular endoscopic submucosal tunnel dissection (ccESTD) 1 2 . However, the survival rate of skin grafts and the prevention of stenosis still need to be improved. Therefore, we used a novel liquid infusion stent for the prevention of esophageal stenosis after ccESTD.

A 67-year-old man diagnosed with wholly circumferential superficial esophageal neoplasm, 27–36 cm from the incisors, underwent double-tunnel ccESTD in our hospital. To fill the long circular artificial ulcer in the esophagus, a novel continuous liquid infusion stent (patent CN 218010622 U) with a 15×8 cm skin graft was used, as described below.

The skin graft was harvested from the right outer thigh of the patient, and the oversleeve-like skin graft was sewn onto the novel continuous liquid infusion stent ( Fig. 1 ). The skin graft with novel continuous liquid infusion stent was placed at the esophageal artificial ulcer. A naso-stent tube attached to the stent was drawn from the nostrils ( Fig. 2 ). A unidirectional valve at the tail of the stent and sustained-release drug storage capsule were designed for temporary storage of liquid ( Fig. 3 ). To test the slow-release function of the stent, methylene blue solution was administered through the naso-stent tube ( Fig. 4 , Video 1 ). Recombinant human epidermal growth factor (rhEGF) solution (100 000 IU) was pumped through the naso-stent tube every 2 days until the stent was removed after 4 weeks. There was no sign of stenosis after 8 weeks, and the main part of the skin graft survived ( Fig. 5 ).

**Fig. 1:**
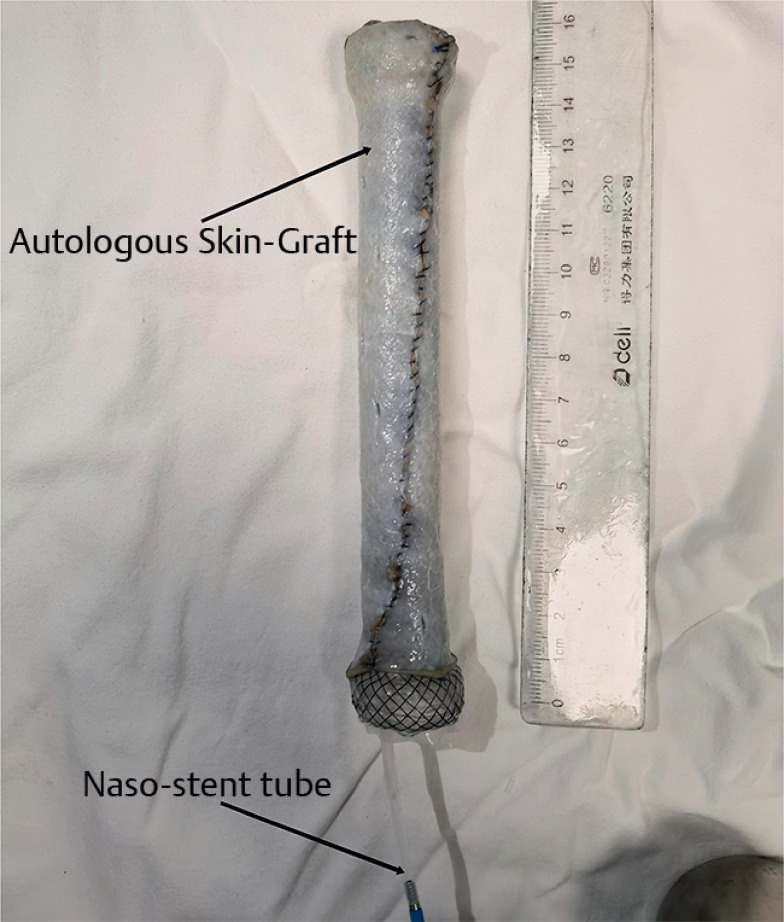
The oversleeve-like skin graft was sewn onto the novel continuous liquid infusion stent.

**Fig. 2:**
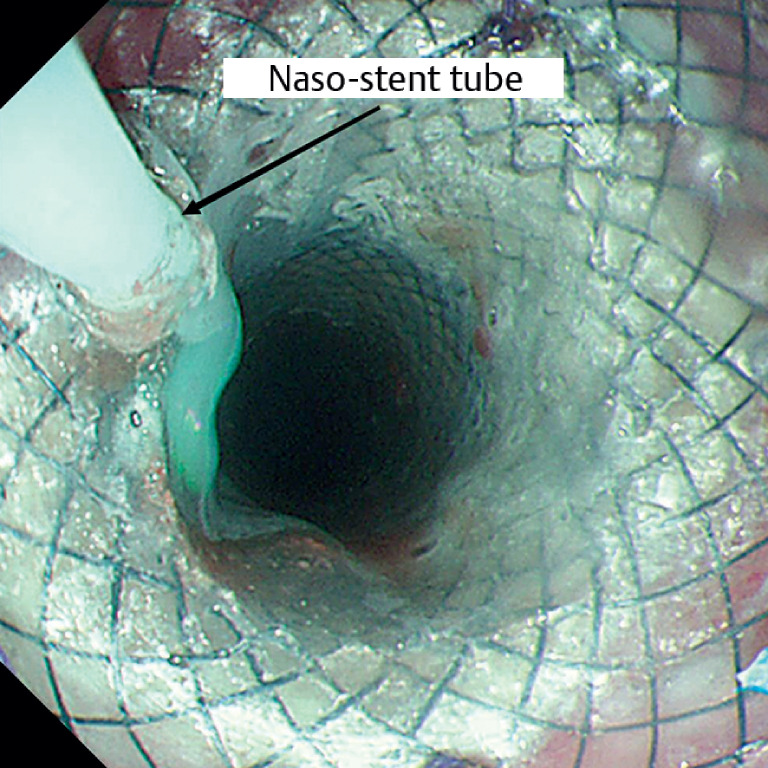
The naso-stent tube attached to the stent was drawn from the nostrils.

**Fig. 3:**
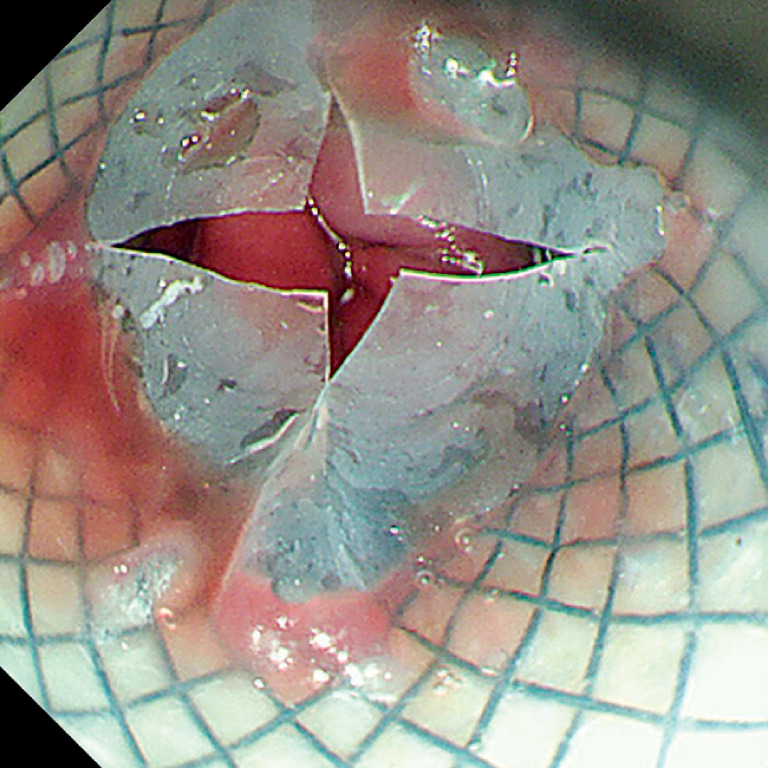
A unidirectional valve at the tail of the stent and sustained-release drug storage capsule were designed for temporary storage of liquid.

**Fig. 4:**
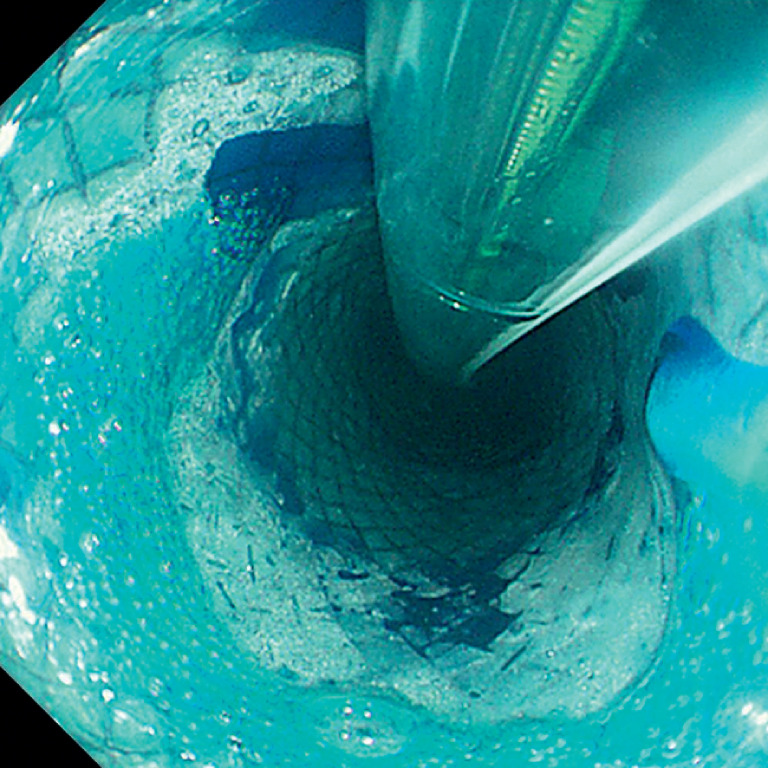
To test the slow-release function of the stent, methylene blue solution was administered through the naso-stent tube.

Autologous skin-grafting surgery with novel continuous liquid infusion stent for the prevention of esophageal stenosis after complete circular endoscopic submucosal tunnel dissection.Video 1

**Fig. 5:**
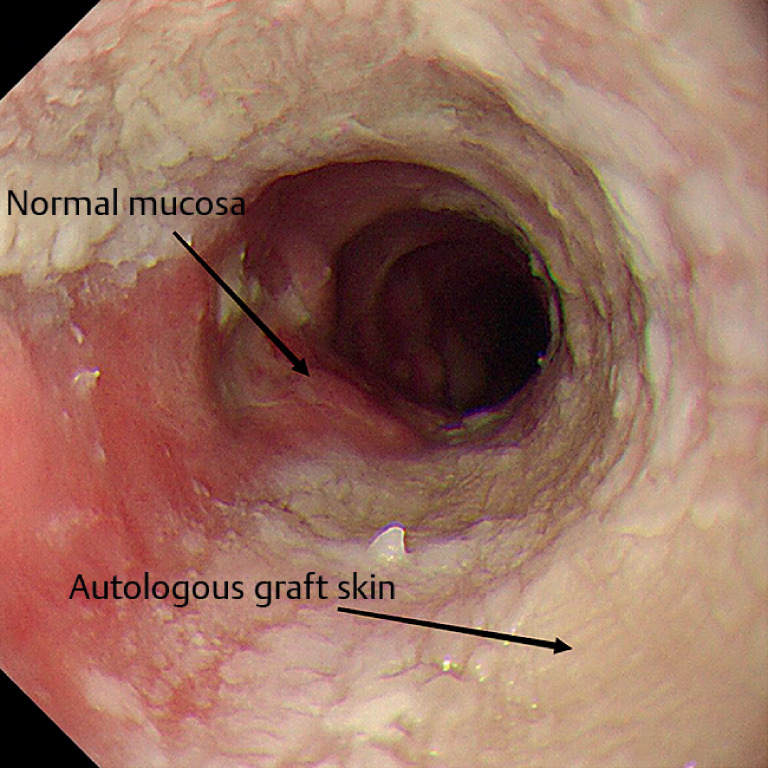
The main part of the skin graft survived after 8 weeks.

To the best of our knowledge, this is the first report of autologous skin-grafting surgery with novel rhEGF infusion stent for prevention of post-ccESTD esophageal stenosis. However, more cases are required to determine the long-term efficacy of this technique in preventing postoperative esophageal stenosis.

Endoscopy_UCTN_Code_TTT_1AO_2AN

## Notes

Endoscopy E-Videos https://eref.thieme.de/e-videos
*E-Videos* is an open access online section of the journal *Endoscopy* , reporting on interesting cases and new techniques in gastroenterological endoscopy. All papers include a high-quality video and are published with a Creative Commons CC-BY license. Endoscopy E-Videos qualify for HINARI discounts and waivers and eligibility is automatically checked during the submission process. We grant 100% waivers to articles whose corresponding authors are based in Group A countries and 50% waivers to those who are based in Group B countries as classified by Research4Life (see: https://www.research4life.org/access/eligibility/ ). This section has its own submission website at https://mc.manuscriptcentral.com/e-videos .

## References

[1] Chai N Zhang W Linghu E Autologous skin-grafting surgery for the prevention of esophageal stenosis after complete circular endoscopic submucosal tunnel dissection Am J Gastroenterol 2018 113 938 10.1038/s41395-018-0142-4 29937541

[2] Chai N Zou J Linghu E Autologous skin-grafting surgery to prevent esophageal stenosis after complete circular endoscopic submucosal tunnel dissection for superficial esophageal neoplasms Am J Gastroenterol 2019 114 822 825 10.14309/ajg.0000000000000169 30882422

